# In vitro comparison of two photostimulable phosphor plate systems for early detection of occlusal dentin caries with and without a sharpening filter

**DOI:** 10.34172/joddd.2020.046

**Published:** 2020-12-06

**Authors:** Roghieh Bardal, Mahshid Mobini, Matin Mirzaee

**Affiliations:** ^1^Department of Oral and Maxillofacial Radiology, Dental Caries Prevention Research Center, Qazvin University of Medical Sciences, Qazvin, Iran; ^2^Student Research Committee, Qazvin University of Medical Sciences, Qazvin, Iran

**Keywords:** Dental caries, Diagnosis of caries, Diagnostic accuracy, Digital radiography, Phosphor plate

## Abstract

**Background.** Dental caries is the most important reason for tooth loss. Clinical examination is the most commonly used technique for occlusal caries diagnosis. The diagnostic power of digital systems is a matter of controversy in this field. The present study aimed to determine the diagnostic accuracy of two photostimulable phosphor plate (PSP) systems for early occlusal dentin caries in vitro.

**Methods.** Sixty-nine extracted molar and premolar teeth were used in this study. The teeth were mounted in triple blocks, and standard radiographs were taken by the Digora and Acteon digital radiographic systems. The original and filter 1-enhanced radiographs were evaluated by two experienced observers twice at an interval of two weeks, and dentin caries was recorded in Tables prepared for the study. The teeth were then sectioned in a buccolingual direction and evaluated under a stereomicroscope. The observers’ reports were compared with microscopic findings as the gold standard. SPSS 23 was used to calculate the kappa coefficient, sensitivity, specificity, and area under the ROC curve (AUC). Statistical significance was set at P<0.05.

**Results.** The internal and the external agreements in both imaging systems were good to excellent. The means of sensitivity, specificity, and AUC in the Acteon system were 34.1, 92.9, and 0.674, with 30.8, 94.8, and 0.659, respectively, in the Digora system.

**Conclusion.** The accuracy of early occlusal caries diagnosis was poor on both systems, and no significant difference was observed between the two systems at a 95% confidence interval. Although the AUC was slightly higher in the original images, there was no significant difference between them; however, due to their high specificity, they can prevent unnecessary treatments in the clinic.

## Introduction

Dental carries, as a progressive bacterial disease, is one of the most common diseases, affecting 95% of the population; it is believed to be the most important reason for tooth loss.^1^ Unfortunately, no accurate and sensitive tool is available to help diagnose dental caries in its initial stages.^2^ Although different techniques, such as analog and digital radiography, transillumination, fluorescence, and tomography, are useful for the diagnosis of incipient caries, radiography is still the most commonly used technique for the diagnosis of caries.^3^ Based on previous studies, 25‒42% of carious lesions remain undetected during clinical examinations without the use of radiographic techniques.^4^ There is controversy over the diagnostic power of radiography for carious lesions.^5^ Some researchers believe that the diagnostic accuracy of E- and F-speed films is similar to that of digital radiography for proximal caries.^6^ Pereira et al^7^ reported that considering the advantages of digital radiography, it appears it is rational to replace digital imaging systems for conventional radiographic systems, even with a diagnostic accuracy similar to that of conventional radiography. Many studies have evaluated the diagnostic power of photostimulable phosphor plate (PSP), CMOS, and CCD digital systems for detecting proximal caries.^8,9^ Contrary to proximal caries, the diagnostic accuracy for occlusal caries is a matter of controversy, despite the fact that determination of the role of caries progression in the enamel and dentin depth is very important for preparing a correct treatment plan.^9^ Therefore, the evaluation of these diagnostic techniques can help dentists select the best diagnostic system for the diagnosis of occlusal caries.^7^ Studies comparing the image quality of phosphor plates with conventional films and the CCD systems have reported a comparable or similar image quality for phosphor plates and a wide dynamic range and higher contrast and resolution with lower exposure doses for PSP.^10^

Controlled clinical and laboratory studies are necessary to determine whether these new digital systems with image enhancement capabilities improve diagnosis, treatment, and prognosis.^11^

Considering the paucity of studies on the subject, the present in vitro study was undertaken to evaluate and compare the accuracy of Digora and Acteon PSP digital systems in the diagnosis of occlusal caries with and without a sharpening filter.

## Methods

**Figure 1:**
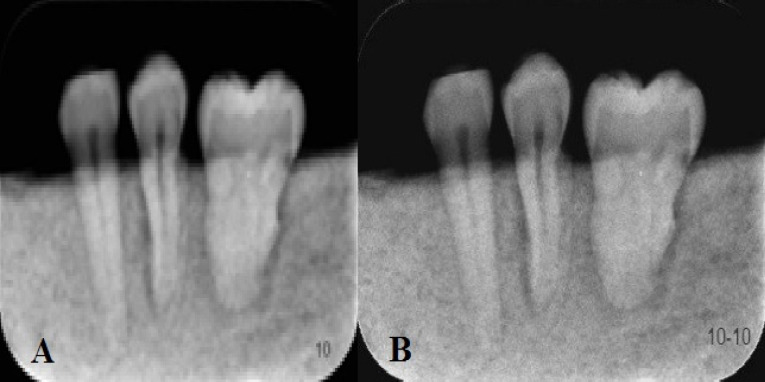
Examples of original (A) and sharped (B) images captured with the Acteon system.

In this experimental study, 69 extracted human molar and premolar teeth, with no visible occlusal cavities, restorations, or Cl V cavities, were evaluated. The teeth were stored in 10% formalin, cleaned with water spray, and dried with an air syringe before being used. In the next stage, the teeth were mounted in blocks of stone and sawdust in rows consisting of three samples. Then the teeth were numbered and underwent a radiographic procedure under standard conditions with the use of Minray unit (Helsinki, Finland) with two different intraoral PSP systems of Digora Optime (Sordex, Helsinki, Finland) and Acteon (Soppro, La Ciotat Cedex France) at kVp=70, mA=8, and an exposure time of 0.2 seconds, already determined in a pilot study. The tooth blocks were placed in a film holder so that the radiographic procedures were carried out under similar geometric conditions. The film-to-tube distance was set at 41 cm, and a piece of Plexiglass was placed between the tooth blocks and the tube to simulate soft tissues. The images were captured with Scanora software and saved with numbers; sharpening filter 1 was then applied to all the images and saved with other numbers (Figure 1). All the images were displayed randomly on a Samsung monitor (Sync Master 740 N) and evaluated by two experienced observers to detect caries twice at an interval of two weeks. The observers were permitted to manipulate images to change the image density and contrast.

The findings reported by the observers were recorded in Tables prepared for this purpose and consisted of the following:

**R0** = no caries

**R1** = dentin caries

Caries odds:

**1** = definitive absence of caries

**2** = caries possibly absent

**3** = cannot be determined

**4** = caries possibly present

**5** = caries definitively present

After radiographic imaging, the tooth samples were retrieved from the blocks and re-mounted in single acrylic resin blocks and numbered. The teeth were sectioned in the buccolingual direction along the vertical axis of each tooth with a Mecatome machine (T201A) (PRESI Co., France) at low speed using a diamond saw (the cutting edge of the blade was made of diamond with a thickness of 0.5 mm). Two or three sections were prepared from each tooth, measuring 1000 µm in thickness.

**Figure 2:**
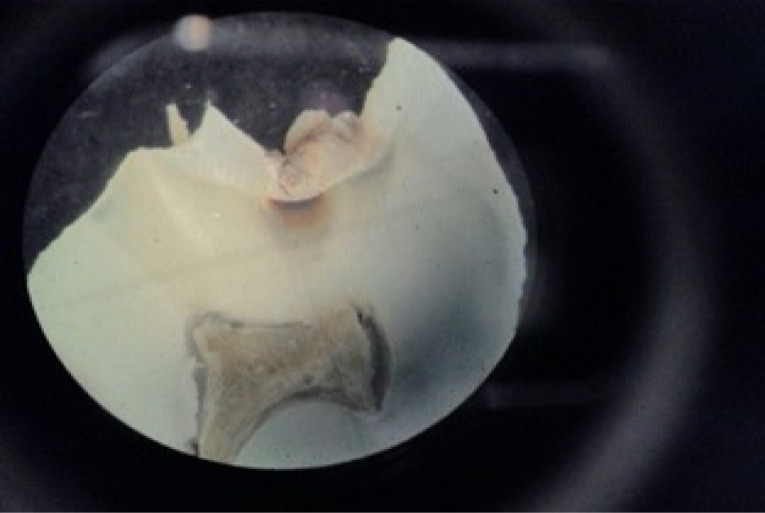
A sample of cross-sections under a stereomicroscope.

Subsequently, the tooth sections were viewed under a stereomicroscope using magnification by a pathologist (Figure 2), and the sound and carious occlusal enamel and dentin were recorded in tables prepared to this end. Finally, the observers’ diagnoses were compared with the histopathological diagnosis as the gold standard.

### Analysis of data

**Figure 3:**
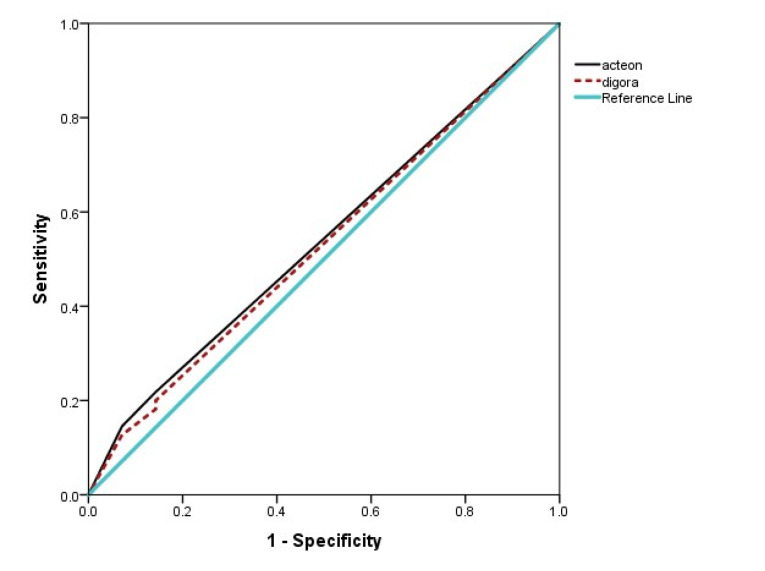
The surface area under the ROC curve for dentin caries separately for each system.

Data were analyzed with SPSS 23. Kappa analysis was used to evaluate intra- and inter-observer agreements. A kappa coefficient of ≥0.8 was considered excellent agreement, with 0.6‒0.79 as good, 0.40‒0.59 as moderate, 0.20‒0.39 as poor, and <0.2 as very poor agreement. To evaluate sensitivity and specificity, a 5-scale table was convened to a 2-scale table so that the values 1 and 2 (caries definitively present and caries possibly present) were considered as the presence of caries, and three other scores were considered as the absence of caries. Z test was used to compare sensitivity and specificity. To evaluate the accuracy, the surface area under the ROC curve (AUC) was used. AUC>0.9 was considered excellent accuracy, with 0.8‒0.9 being considered good, 0.7‒0.8 as moderate, and 0.6‒0.7 as poor accuracy (Figure 3).

## Results

Histopathological evaluation of 69 teeth in the present study showed that 14 teeth (20.3%) were sound, with 25 teeth (46.2%) and 30 teeth (43.5%) exhibiting enamel and dentin caries, respectively. Intra- and inter-observer agreement results are presented in Table 1. Intra-observer agreement for the first observer was between 0.541 and 1.00, with 0.641 and 1.00 in the second observer. The lowest agreement was related to the Acteon system in the sound teeth. The inter-observer agreement was between 0.451 and 1.00. The lowest agreement was related to the first observation of sound teeth in the Acteon system (Table 1).

**Table 1:** Kappa coefficients separately for each system irrespective of caries

		**Digora**				**Acteon**			
		**Sharp**		**Un-sharp**		**Sharp**		**Un-sharp**	
		**Sound**	**Caries**	**Sound**	**Caries**	**Sound**	**Caries**	**Sound**	**Caries**
Intra-observer agreement	First observer	0.720							
	Second observer	1.00	0.640	1.00	0.737	0.541^*^	0.700	0.541*	0.795
	First observation		0.931	1.00	0.800	0.641	0.927	0.641	
	Second observation	0.720							0.769
Inter-observer agreement	First observer	1.00	0.736	1.00	0.877	0.451*	0.850	0.451*	
	Second observer		0.848	1.00	0.780		0.782	1.00	
	First observation					1.00			0.659
	Second observation								0.769

The sensitivity of both systems was 28.3 to 34.1, with a specificity of 92.9‒94.8 (Table 2).

**Table 2:** The means of sensitivity, specificity, and the surface area under the ROC curve separately for each system in dentin caries

	**Digora**		**Acteon**	
	**Sharp**	**Unsharp**	**Sharp**	**Unsharp**
Dentin sensitivity	28.3	30.8	32.5	34.1
Dentin specificity	94.2	94.8	92.9	92.9
Dentin AUC (CI)	0.645 (0.578,0.713)	0.659 (0.592,0.762)	0.649 (0.581,0.716)	0.674 (0.608,0.704)

The accuracy [(true positive + true negative)/total observations] of both systems in two filtering modes was measured and compared with the AUC and the related confidence intervals Table 2); the difference in AUC was not significant [0.645 (CI: 0.578‒0.713) vs. 0.659 (CI: 0.592‒0.762), 0.649 (CI: 0.581‒0.716) and 0.674 (CI: 0.608‒0.704)]. According to Table 3, this difference was not significant concerning the observer.

**Table 3:** The surface area under the ROC curve for dentin caries separately for each system

		**Digora, AUC (CI)**		**Acteon, AUC (CI)**	
		**Sharp**	**Unsharp**	**Sharp**	**Unsharp**
First observer	First observation	0.654 (0.519, 0.789)	0.667 (0.533, 0.801)	0.637 (0.501, 0.733)	0.711 (0.582, 0.840)
	Second observation	0.609 (0.472, 0.747)	0.643 (0.507, 0.779)	0.626 (0.489, 0.763)	0.658 (0.523, 0.792)
Second observer	First observation	0.659 (0.524, 0.794)	0.690 (0.559, 0.822)	0.673 (0.539, 0.807)	0.685 (0.553, 0.818)
	Second observation	0.658 (0.523, 0.792)	0.638 (0.502, 0.774)	0.659 (0.524, 0.794)	0.642 (0.506, 0.778)

## Discussion

Several studies have evaluated the effects of manipulating digital images and different filters on the diagnostic accuracy of images, with different results. Based on some studies, the manipulation of images and the use of different filters such as ‘sharp’ does not affect on increasing the caries diagnostic power of digital radiographic techniques.^12^ Studies by Belem et al^12^ and Kositbowornchai et al^13^ reported such a result. In the present study, the observers were allowed to manipulate images to change contrast, density, and magnification. Besides, the effect of sharpening filter 1 on the diagnosis of occlusal caries was evaluated.

Intra- and inter-observer agreement in both systems for original and sharpened images was good to excellent, except for star-marked sound surfaces in the Acteon system (Table 1). The agreement rates in the study carried out by Rocha et al^14^ were 0.51 and 0.31 in dental students and radiologists. The kappa coefficients in the study carried out by Shams et al^15^ in third-year students, last-year students, postgraduate radiology students, and general dental practitioners were 0.002, 0.073, 0.271, and 0.03, respectively, which are lower in both studies compared to the present study.

The differences in internal and external agreement rates between different studies might be attributed to the following: 1) the experience of the observers with digital systems; 2) the diagnostic capabilities of the observers concerning caries; 3) the time interval between the observations; 4) the number of observers.

Based on Table 2, the sensitivity of both systems was 28.3 to 34.1, with no significant difference between the two systems (*P* > 0.3); the accuracy of early diagnosis of occlusal caries was poor in both systems, and no significant difference was observed between the two systems at 95% confidence interval. These results were similar to some previous studies.^7,16-18^

Although sensitivity and the AUC were slightly higher in the ‘unsharp’ mode compared to the ‘sharp’ mode, there was no significant difference between them. Filters that sharpen an image enhance boundaries with high-frequency noise removal; therefore, sharpening filters remove grey scales that might have a diagnostic value in the detection of incipient caries.

In Shokri et al^19^ study, the sensitivity and accuracy of filtered images were significantly higher than original images; this difference was higher in superficial images. Caries in this study was artificially created chemically and had more regular outlines; this affects caries detection on radiographs.

Experience with digital systems strongly affects the results of such studies.^20^ Shams et al^15^ evaluated the effect of experience and education on the diagnosis of proximal caries in 2015 and concluded that although experience and knowledge are effective in improving the accuracy of detecting caries on digital images, it does not increase the diagnostic accuracy to the optimal level. Mileman et al^21^ and Rocha et al^14^ evaluated the effect of experience on the diagnostic accuracy of occlusal and proximal caries and reported that inexperienced students exhibited the highest false positive rate, and radiologists exhibited the highest false negative rate in their reports. In the present study, two radiologists evaluated the images, and consistent with the studies above, there were more false-negative reports than false-positive reports.

In studies by Wenzel et al,^22^ Hintze et al,^23^ Rocha et al,^14^ Hintze,^24^ Yalçinkaya et al,^20^ and Tarım Ertas et al^25^ to compare different digital systems and conventional films, it was concluded that there was no significant difference between the accuracy of different radiographic systems, consistent with the results of the present study. Tyndall et al^26^ evaluated the effect of manipulating the contrast and density of digital images on their diagnostic efficacy and concluded that the manipulated images exhibited significantly lower accuracy than conventional radiographs and un-manipulated digital images. They used a CCD digital system (Sidexis) for imaging procedures and reported that Sidexis systems use a special processing filter, which improves the images before displaying them on the monitor, and this processing filter might interfere with changes in contrast and illumination, decreasing the diagnostic accuracy in manipulated images. Also, inadequate training and incorrect use of the software program used to manipulate digital images are considered other reasons for a decrease in the accuracy of the above-mentioned manipulated images.

The mean surface area under the ROC curve for the occlusal surface in the study carried out by Wenzel et al^22^ was 0.873 for different digital systems, which is higher than the present study. In the study by Wenzel et al,^22^ teeth with occlusal cavities were not excluded from the study.

The mean surface area under the ROC curve in the study carried out by Hintze^24^ in different digital systems was approximately 0.7, which is higher than that in the present study. In this study, enamel and dentin caries on the proximal surface and dentin caries on occlusal surfaces were evaluated, and both surfaces were evaluated simultaneously to calculate diagnostic accuracy; however, in the present study, proximal caries was not evaluated.

## Conclusion

The present in vitro study, with small sample size, showed that the diagnostic accuracy of two PSP sensors of Acteon and Digora systems was the same for the diagnosis of occlusal caries. Although both systems exhibited low sensitivity for the detection of early dentin caries, their high specificity can prevent unnecessary procedures in the clinic. There was no significant difference between images enhanced with enhancement filter 1 and original images.

## Authors’ Contributions

Concept or design of the study: RB. Data acquisition, analysis, or interpretation: MM, MM. Writing the manuscript: RB. Critical revision of the article: RB, MM. Final approval of the manuscript: RB, MM, MM. Overall responsibility: RB.

## Acknowledgements

The authors would like to thank all the colleagues who sincerely offered their assistance for carrying out this study.

## Funding

None.

## Competing Interests

The authors declare no competing interests with regards to the authorship and publication of this article.

## Ethical Approval

Not applicable.
